# Near-Infrared Spectral MEMS Gas Sensor for Multi-Component Food Gas Detection

**DOI:** 10.3390/mi16020135

**Published:** 2025-01-24

**Authors:** Xiaojian Yan, Yao Tan, Yi Wang, Gongdai Chen, Weigao Xia, Gang Zhou, Hongliang Luo, Hao Liu, Tianxun Gong, Xiaosheng Zhang

**Affiliations:** 1 School of Integrated Circuit Science and Engineering, University of Electronic Science and Technology of China, Chengdu 611731, China; yanxj@changhong.com (X.Y.); yntanyao@163.com (Y.T.); txgong@uestc.edu.cn (T.G.); 2 Panovasic Technology Co., Ltd., Chengdu 610041, China; yi4.wang@changhong.com (Y.W.); gongdai.chen@changhong.com (G.C.); weigao.xia@changhong.com (W.X.); zhoug@changhong.com (G.Z.); hongliang1.luo@changhong.com (H.L.); hao3.liu@changhong.com (H.L.)

**Keywords:** MEMS, micro-electromechanical systems, near-infrared spectroscopy, gas sensor

## Abstract

The complex application environments of gas detection, such as in industrial process monitoring and control, atmospheric and environmental monitoring, and food safety, require real-time and online high-sensitivity gas detection, as well as the accurate identification and quantitative analysis of gas samples. Despite the progress in gas analysis and detection methods, high-precision and high-sensitivity detection requirements for target gases of multiple components in mixed gases are still challenging. Here, we demonstrate a micro-electromechanical system (MEMS) with near-infrared (NIR) spectral gas detection technology and spectral model training, which is used to improve the detection and classification of multi-component gases in food. During blind sample testing, the NIR spectral gas sensor demonstrated over 90% accuracy in identifying mixed gases, as well as achieving the classification of ethanol concentration. We envision that our design strategy of an NIR spectral gas sensor could enhance the gas detection and distinguishing ability under the conditions of background gas interference and cross-interference in multi-component detection.

## 1. Introduction

With the increasing demand for human health and life quality, the rapid detection of foods’ freshness based on food odor is a rapidly developing field [1,2,3]. Especially in the fields of food preservation [4,5], perfume formulation, and environmental protection, the usual approach is to use a combination of gas sensors with different gas sensitivities to detect different types of gases, but this often leads to cross-interference response problems between chips with different gas-sensitive principles. Although progress in many gas analysis and detection methods has been made, such as in gas chromatography-mass spectrometry, semiconductor gas sensor detection, and electrochemical gas sensor detection, and so on [6,7,8,9,10], conventional spectrometers are usually heavy, expensive, and demand high installation and maintenance costs, limiting the broader applicability of these methods [11,12]. Constructing such a gas sensor in an integrated miniaturized format to achieve high-precision and high-sensitivity detection of target gases of multiple components in mixed gases is still challenging [13,14,15,16,17,18]. For this reason, miniaturized spectrometers have gained both academic and industrial momentum in the last few years with many proposals using different technologies, where MEMS technology is a strong candidate [19,20,21].

While mid-infrared (MIR) gas sensors are well-established, the photodetectors in MIR gas sensors usually face challenges, such a higher noise and slower response times than NIR sensors. Additionally, MIR gas sensors typically require cooling, which further complicates their use [22,23,24,25,26,27]. However, in the context of low-cost gas detection, despite their lower sensitivity (i.e., it is less effective for detecting gases at concentrations below 0.1%w), NIR gas sensors feature distinctive characteristics, such as not requiring the injection of high-purity nitrogen to obtain a baseline under non-absorption conditions, while their ability to carry out long-distance measurements reduces the restrictions on the test environment; as such, they have received substantial interest. The working principle of NIR gas sensors relies on the unique absorption fingerprints in the NIR spectral band (780–2526 nm) of gas molecules, and the frequency and harmonic frequencies of this regime match the vibration frequencies of the hydrogen-containing groups in gas molecules; the different organic molecules of gases can experience a unique resonant absorption with the NIR light at specific wavelength positions, further allowing us to distinguish between mixed gases [28,29]. With the progress of MEMS technology, the opto-electromechanical system of NIR spectrometers can be integrated into a chip, achieving compactness and reduced cost, and reliable MEMS NIR spectral gas sensors with a miniaturized size are highly desirable [30].

In fact, quantitative analysis of gas spectra using an NIR spectrometer has been reported successfully in the literature. In addition to the MEMS NIR spectrometers’ portability, which enables their use in unconventional applications and environments, they do not interact with the measured gases and can detect multiple gases and identify their concentrations simultaneously. Manzardo et al. [31] first reported a MEMS moving mirror which attached to a comb-drive actuator to implement the function of one movable arm of a Michelson interferometer. After that, various gas sensors taking advantage of MEMS core components assembled with discrete optical elements were developed. The use of a silicon Bragg reflector in a Michelson interferometer was reported by Saadany et al. [32], where the use of the Bragg mirrors reduced the device footprint, thus overcoming the extra space needed by the technology to metallize vertical surfaces. A miniaturized Fourier transform infrared (FT-IR) spectrometer with the resolution and the SNR limits of miniaturization was also reported by Erfan et al. [33]: it performed a clear identification of the different compositions of a mixed gas as acetylene (C_2_H_2_), carbon dioxide (CO_2_), and water vapor (H_2_O). Although the detection of multiple gases simultaneously is one of the important advantages of using infrared spectroscopy with a wide spectral range in gas sensing applications, and the MEMS NIR spectral gas sensors achieved lightweight gas detection, there are still the issues of the rapidity, high sensitivity, and accuracy of the gas detection module of MEMS NIR spectral gas sensors, restricting their wide usage in optical gas detection (the absorption intensity in the NIR band is relatively low). It is noticed that, in NIR spectroscopy analysis, there is a certain functional relationship between the composition and structure of the gas sample and the near-infrared spectrum. Therefore, using chemometric methods would establish a model between the chemical values and the spectral information. The established models in the software could be used for quick multi-component detection and for the performance enhancement of mixed gas samples information using the hardware.

Herein, to address the problem of low gas density and the difficulty of capturing spectral absorption information, we employed a silicon wafer surface treatment method to design a Fabry–Perot cavity with six layers of optical stacked films in the hardware part to increase the optical path and enhance the gas spectral absorption information. In the software part, spectral preprocessing and spectral modeling algorithms were combined to effectively differentiate mixed gases and perform high-precision and high-sensitivity gas detection. Using three-dimensional stacked packaging to reduce the overall volume of the microsystem, we realized a MEMS NIR spectral gas sensor with a fingertip size. Notably, the presented MEMS NIR spectral gas sensor displays selective and sensitive detection within seconds toward ethanol, Korean kimchi, and durian pulp, which are easy to taint and difficult to distinguish. Moreover, the platform can also be used for the simultaneous detection of the concentration of ethanol and is applicable to practical household and industrial environments because of its portability, small size, ethanol detection limit of around 369 ppm. The MEMS NIR spectral gas sensor provides novel perspectives for the performance enhancement of gas sensors, which enables the creation of NIR gas sensors with high sensitivity, a fast response/recovery, and low power consumption, which are promising traits for mass production and commercialization.

## 2. Detection System and Methods

### 2.1. Fabrication of MEMS Fabry–Perot Cavity

**Figure 1:**
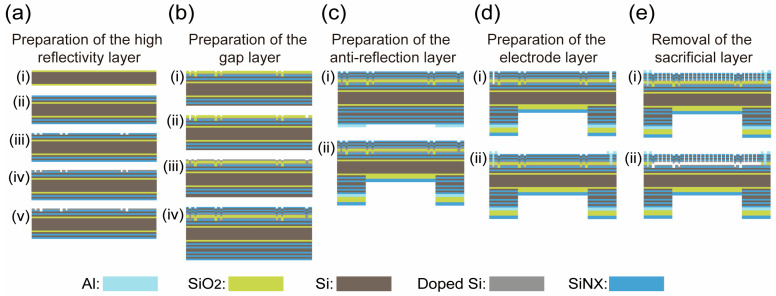
Appearance of the NIR spectral gas sensor. (**a**) The step for the preparation of the high reflectivity layer. (**b**) The step for the preparation of the gap layer. (**c**) The step for the preparation of the anti-reflection layer. (**d**) The step for the preparation of the electrode layer. (**e**) The step for the removal of the sacrificial layer.

Here, we adopted the silicon wafer surface treatment method to process the Fabry–Perot cavity. Firstly, we used the chemical vapor deposition (CVD) method to grow a SiO_2_ buffer layer film with a thickness of 1/4 the center wavelength (around 1700 nm) in 6 inches of silicon wafer with a thickness of 450 μm, as shown in Figure 1a(i). Then, according to the high and low refractive index matching method of DBR (distributed Bragg reflector) film, polysilicon (with a refractive index of 3.5) was selected as the high refractive index material, and SiN_x_ was used as the low refractive index material (with a refractive index of 2.0), and the CVD process was used to alternately grow a polysilicon/SiN_x_ film (with 6 layers optical stacked films) with a thickness of 1700 nm, as shown in Figure 1a(ii). Next, we performed photolithography on the basis of the SiN_x_ film to form an electrode pattern, as shown in Figure 1a(iii). We then continued to grow a polysilicon film on this basis and doped it, as shown in Figure 1a(iv). The doped polysilicon film, as the last layer of the DBR film, needed to be photolithographically processed again to design an electrode pattern, as shown in Figure 1a(v).

So far, the first high reflectivity layer of the Fabry–Perot cavity has been manufactured. The next step is to design the gap layer. We used the plasma-enhanced CVD (PECVD) process to grow a 1950 nm thicker single-sided SiO_2_, as shown in Figure 1b(i). At the same time, since an electrostatically tuned positive and negative electrode connection area was designed below the gap layer, it is necessary to perform photolithography patterning in the corresponding electrode area of the gap layer, as shown in Figure 1b(ii). After the gap layer is manufactured, we used low-pressure CVD (LPCVD) to grow a polysilicon film with a thickness of 1700 nm above the gap layer and then performed ion implantation to form a film which has a common potential with the electrode area below the gap layer, as shown in Figure 1b(iii). After the polysilicon film above the gap layer was grown, we continued to use the LPCVD process to alternately grow a high and low refractive index SiN_x_/polysilicon film (Panovasic Technology Co., Ltd, Chengdu, China), as shown in Figure 1b(iv).

Next, in order to design a light shielding on the back to avoid the interference from stray light, an Al film with the thickness of 800 nm was grown on the back, and a hole was photolithographically opened at the position of the device’s aperture, as shown in Figure 1c(i). In order to improve the transmittance of the device, an optical anti-reflection film should be designed to improve the transmittance of the Fabry–Perot cavity, as shown in Figure 1c(ii).

After the anti-reflection layer was made, the entire wafer needed to be turned over to continue making the front structure of the Fabry–Perot cavity. First, the electrode contact hole needed to be made, as shown in Figure 1d(i). Then, Al was sputtered into a film and the electrode production was completed using photolithography, as shown in Figure 1b(ii). After the electrode layer was made, the sacrificial layer needed to be released. Here, we used the dry etching process to evenly arrange release holes on the 6 layers of optical stacked films above the sacrificial layer, as shown in Figure 1e(i). After the release holes were made (the diameter of the release holes was around 3 μm), the gap layer of the Fabry–Perot cavity could be etched by wet etching technology to form a gap layer (the thickness of gap layer was around 22 μm). As a result, the device was completed, as shown in Figure 1e(ii).

### 2.2. Construction of the Gas Detection System

**Figure 2:**
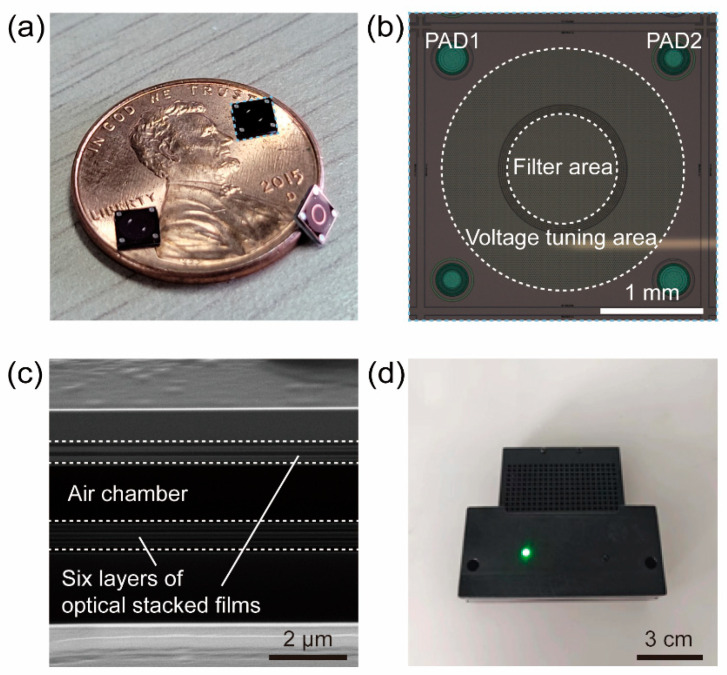
Appearance of the internal chip of the NIR spectral gas sensor. (**a**) The image of the NIR MEMS Fabry–Perot cavity spectral sensing chip. (**b**) The specific composition of the spectral sensing chip. (**c**) Side SEM image of the spectral sensing chip. (**d**) The appearance of the NIR spectral gas sensor.

**Figure 3:**
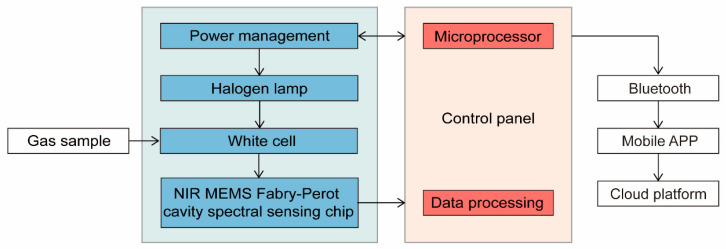
The diagram of the system of the NIR spectral gas sensor. The blue part represents the hardware part of the system, which is used to make the gas sample in the White cell produce spectral absorption and to detect the light signal absorbed by the gas molecules. The red part represents the peripheral circuit of the system, which is used to implement power management, signal processing, and sending information to the software part by Bluetooth.

After the preparation of the NIR MEMS Fabry–Perot cavity spectral sensing chip was completed (the images are shown in Figure 2), we constructed the hardware system of the NIR spectral gas sensor, which consisted of the NIR MEMS Fabry–Perot cavity spectral sensing chip, a collimating lens, a halogen lamp, and a White cell, as shown in Figure 3.

As shown in Figure 2b, the spectral sensing chip consisted of three parts, namely the pin pads, the filter area, and the voltage tuning area. Specifically, the four pin pads (named PAD1 and PAD2) were used to drive the chip by applying voltage, while the filter area refers to the area through which the light passes, and the Voltage tuning area refers to the area in which the voltage forms an electrostatic field. When the chip is working, the upper six layers of the optical stacked films with different refractive indexes will sink under the action of the electrostatic field formed by the voltage difference loaded between PAD1 and PAD2 (the details are shown in Figure 2c), changing the height of the air chamber and thereby achieving the effect of filtering light of different wavelengths.

**Figure 4:**
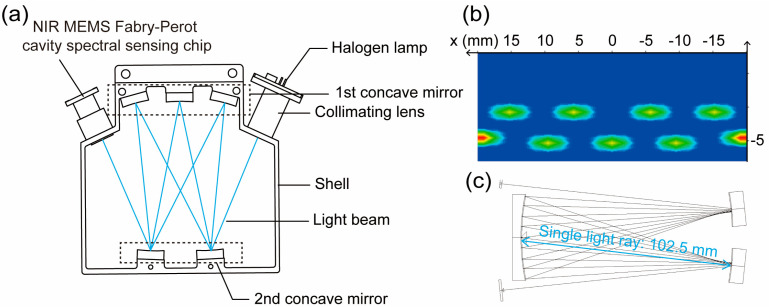
The diagram of the White cell and the effect of the light beam reflecting back and forth multiple times between the first concave mirror and the second concave mirror of the White cell. (**a**) The diagram of the White cell: the blue line represents the light beam that multiple reflections in the White cell. (**b**) The illumination spot on the first concave mirror. (**c**) The actual gas absorption light beam between the first concave mirror and the second concave mirror. The single light ray, indicated by the blue line, is 102.5 mm in length.

In the gas detection and distinguishing process of the NIR spectral gas sensor, the spectral absorption of gas is based on Lambert–Beer’s law, as follows:

*A* = lg(1/*T*) = *kcd* (1)

where *A* is the absorbance, *T* is the translucency (*T = l/l*_0_, where *l* is the outgoing light intensity; *l*_0_ is the incident light intensity.), *k* is the molar absorption coefficient, *c* is the concentration of the absorbing substance, and *d* is the optical path length. From Equation (1), we obtain the monotone increase in *A* with *d*, that is, increasing the *d* would be another effective method to improve detection sensitivity with selected absorption spectral lines, which could be achieved by using multiple reflection technology and cavity-enhanced absorption spectroscopy (CEAS). As a multiple reflection absorption cell, the White cell allows the light beam to reflect back and forth multiple times between high reflectivity mirrors (Figure 4a), allowing the *d* to be increased to several hundred meters (Figure 4b). However, increasing the *d* limits the dynamic range of measurement (i.e.,the maximum measurable gas concentration) and severe interference noise. Therefore, software-based filtering technology and mathematical model training recognition are applied to improve the maximum measurable gas concentration. Here, the effective optical path length was designed and calculated using Light Tools (version 8.4). In the simulation, the divergence angle of the halogen lamp was set to 0 degrees, and we modeled the path of light as it reflected multiple times between mirrors within the White cell. As shown in Figure 4c, the length of a single light ray, indicated by the blue line, is 102.5 mm. The light ray travels back and forth 20 times within the White cell, resulting in an effective *d* of approximately 20 × 102.5 mm = 2050 mm.

The software system of the NIR spectral gas sensor consists of a sensor sampling algorithm, embedded software, an Android client, and a spectral data processing platform. The sensor sampling algorithm and embedded software realize high-precision sampling, fast calculation, and intelligent control of the NIR spectral gas sensor. The gas detection module is connected to the Android client via Bluetooth, and the Android client can realize functions, such as collection, analysis, and spectral data display. Furthermore, the Android client collects and interconnects with the spectral data processing on the cloud platform, and the collected data is stored on the cloud platform, which performed various functions, such as data analysis, platform modeling, and local model importing.

### 2.3. Performance Test of the NIR MEMS Fabry–Perot Cavity Spectral Sensing Chip

**Figure 5:**
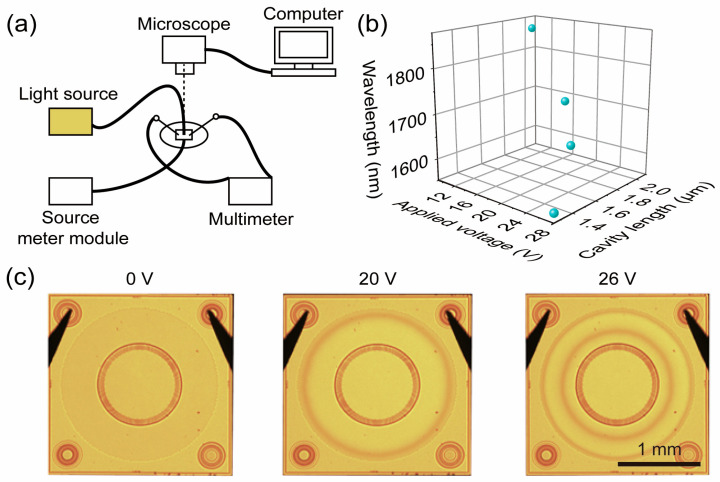
Performance test of the NIR MEMS Fabry–Perot cavity spectral sensing chip. (**a**) Test schematic diagram, including the test of the transmittance using a monochromator as the light source and the test of the electrostatic tuning using a cold light source as the light source. (**b**) The relationship of the change in the Fabry–Perot cavity length and the central transmission wavelength of the maximum transmittance with the change in the applied voltage. (**c**) The state of the NIR MEMS Fabry–Perot cavity spectral sensing chip under different applied voltages.

The NIR MEMS Fabry–Perot cavity spectral sensing chip has the effect of filtering light of a specific wavelength. The schematic diagram of the test system for this performance is shown in Figure 5a. Here, for testing the transmittance of different wavelengths, we used a monochromator as the light source. Since the NIR MEMS Fabry–Perot cavity spectral sensing chip is voltage-modulated, when different voltages are applied to the device, this will affect the Fabry–Perot cavity length and, furthermore, the central transmission wavelength of the maximum transmittance. As shown in Figure 5b, which shows the different optical transmittance characteristics under 4 different voltages, the wavelength corresponding to the 26 V voltage exhibits the highest transmittance, which is caused by the length of the MEMS Fabry–Perot cavity.

In addition, we also designed a test system to check whether the device could be pressurized and controlled, that is, whether the distance can be tuned. The test system is shown in Figure 5a. Here, for testing the electrostatic tuning of different voltages, we used a cold light source as the light source. As shown in the Figure 5c, as the applied voltage increases, the cavity length of the Fabry–Perot interferometer decreases, creating wedge-shaped gaps with varying wedge angles at the edges. Under optical microscope observation, the wavelength of the microscope’s light source forms an equal thickness interference phenomenon at the wedge gap position, resulting in distinct interference rings of different shapes.

### 2.4. Response and Recovery Time of the MEMS NIR Spectral Gas Sensor

**Figure 6:**
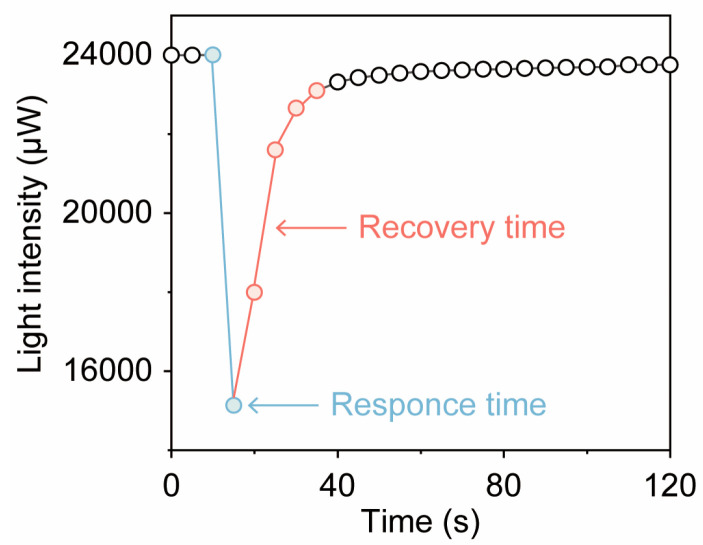
Relationship between the test time after the water vapor sample with a relative humidity (RH) of 80% enters the test environment (with RH maintained at 50% ± 10%) and the light intensity detected by the sensor. The blue line represents the sensor’s response time, and the red line represents the sensor’s recovery time.

The MEMS NIR spectral gas sensor operates based on optical principles, meaning that its response time depends primarily on the time required to scan the spectrum and the gas diffusion time. When the gas diffusion time is not considered, the response time is mainly determined by the spectrum scanning time, which is influenced by the sensor’s sampling frequency and the data transmission time. Here, we measured the relationship between the time elapsed after the water vapor sample, with a relative humidity (RH) of 80%, entered the test environment (with RH maintained at 50% ± 10%) and the light intensity recorded by the sensor at 1543 nm wavelength (Figure 6). Figure 6 shows a sudden drop in light intensity. We considered this sudden drop in light intensity to be the response time of the sensor, which is about 5–6 s, and the slow rise of the light intensity after a sudden drop is considered as the recovery time, which is about 20–25 s.

### 2.5. Configuration of Gas Samples

Gas samples are divided into three categories, namely ethanol (AR), the smell of Korean kimchi, and the smell of durian pulp. Among them, ethanol is a characteristic smell produced by fruit corruption, Korean kimchi is an odor source that mixes easily the refrigerator, and the smell of durian pulp is an odor source with a characteristic smell but one not caused by food corruption.

During the gas sample collection and classification process, the above three representative gas samples were collected as single samples, pairwise random combination samples, and all combination sample. Specifically, there were 8 combinations, namely ethanol, Korean kimchi, durian pulp, ethanol–Korean kimchi, ethanol–durian pulp, Korean kimchi–durian pulp, ethanol–Korean kimchi–durian pulp, and air. Here, air was designed as a blank sample because it does not have a smell.

### 2.6. Gas Samples Classification and Identification Experiment

The experiment was carried out in a sealed 52 L transparent plastic box, with one gas sample corresponding to one transparent plastic box. The NIR spectral gas sensor and a gas sample were placed in a same transparent plastic box.

During the experiment, we put the odor source in the box for 1 min before starting the test to ensure that the gas sample in the box was evenly diffused. The volume of ethanol was 0.2 mL, the mass of the Korean kimchi was 5 g, and the mass of the durian pulp is 20 g, in order to ensure that the concentrations of the gas samples were the same.

After the experiment was completed, we opened the box lid for 2 h to remove the odor before conducting the next test. At the same time, the experimental environment was kept well ventilated to prevent odor remaining in the environment.

### 2.7. Ethanol Concentration Grading Experiment

The experiment was carried out in a sealed 52 L transparent plastic box, in which the NIR spectral gas sensor and the electronic balance were placed. During the experiment, different volumes of ethanol (0.05 mL, 0.1 mL, 0.15 mL, 0.2 mL, 0.25 mL, 0.3 mL, 0.35 mL, and 0.4 mL) were injected into a petri dish on the tray of electronic balance, and then the transparent plastic box was quickly sealed. It can be observed that the data displayed on the electronic balance gradually decreased because of the continuous evaporation of ethanol. The spectrum of the gas sample in the transparent plastic box was collected within 1 min after the ethanol had completed its volatilization and diffusion, in order to ensure that the concentrations of the gas samples were the same. After the experiment was completed, we opened the box lid for 2 h to remove the odor before conducting the next test. At the same time, the experimental environment was kept ventilated to prevent the odor remaining in the environment.

### 2.8. Spectral Data Preprocessing and Model Training

The model was trained by combining different spectral data preprocessing methods with multiple qualitative modeling method hyperparameters. Spectral data preprocessing mainly used smoothing, derivation, baseline calibration, detrending, standard normal variate (SNV) transformation, normalization, logarithm, etc. The qualitative modeling method mainly uses linear discriminant analysis and support vector machines with different kernel functions. The cross-validation method is used to optimize the model parameters, and the optimized model is used for blind sample testing.

## 3. Results and Discussions

### 3.1. Classification and Identification of Mixed Gases

**Figure 7:**
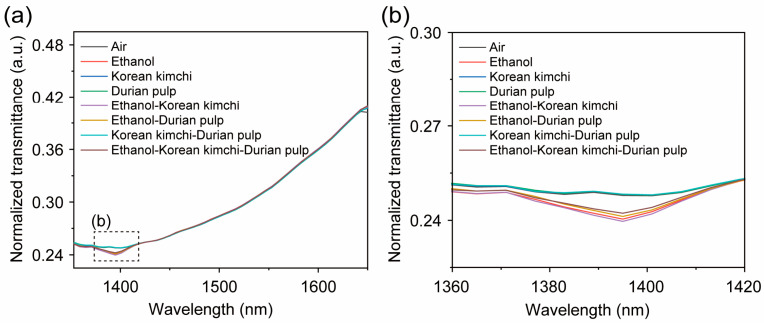
The near infrared spectra of 8 mixed gas samples. (**a**) The normalized transmittance of 8 mixed gas samples in the 1350 to 1650 nm wavelength range. (**b**) The normalized transmittance of 8 mixed gas samples in the 1360 to 1420 nm wavelength range. The air in this test is set as the blank sample; of the 166 mixed gas samples, we extracted 1 gas sample from each mixed gas samples to form this near-infrared spectrum.

In order to avoid the interference of the collinearity of spectral variables, we adopted the sample set division method (the Kennard–Stone algorithm) to divide the 166 samples into training set samples and blind test set samples at a ratio of around 3:1. A total of 116 training set samples were used to build the calibration model, and 50 samples were used as the blind test set to evaluate the performance of the calibration model. This is because the spectral information of the samples is affected by various factors, such as the samples’ density, particle size, water content, and environmental temperature and humidity. Before building a machine learning correction model, the spectral information of samples needs to be preprocessed to improve the correlation between the spectral information and the chemical value and improve the prediction performance of the model. Here, we used the normalization method to process samples of the ethanol, the smell of Korean kimchi, and the smell of durian pulp, as well as their mixed gas samples. The normalized spectral information of each gas sample is shown in Figure 7a.

**Figure 8:**
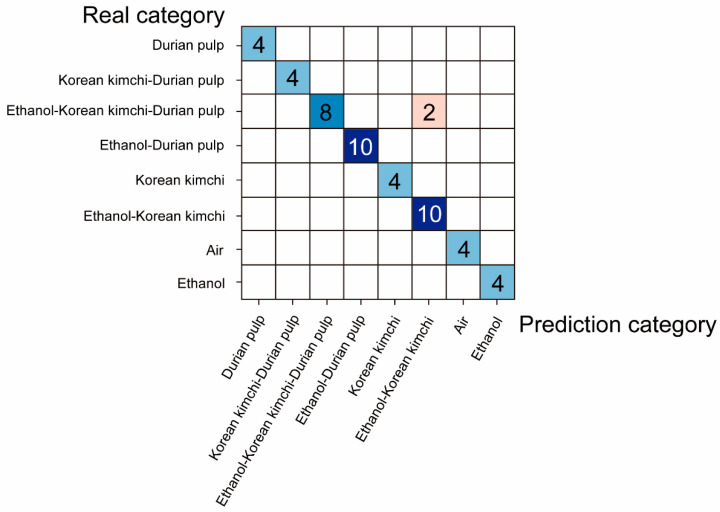
The confusion matrix of the mixed gas samples blind test. The blue block indicates that the prediction category is consistent with the real category, and the red block indicates that the prediction category is inconsistent with the real category. The darker the color block, the more correct samples are predicted.

From Figure 7b, we can find that, compared with other gas samples, the gas samples containing Korean kimchi had a more obvious difference in spectral information between the wavelengths of 1375–1425 nm, but there was no obvious difference in the other wavelengths. This may be because the sensitivity of the NIR spectral gas sensor is relatively low, resulting in it struggling to distinguish multi-component gas samples, so it is necessary to combine the NIR spectral gas sensor with machine learning methods. Table 1 shows the accuracy of different machine learning methods for distinguishing multi-component gas samples. As in Table 1, we used normalized spectral preprocessing combined with the quadratic kernel function support vector machine (SVM) algorithm, which demonstrated a cross-validation accuracy of 91.4% and a blind test accuracy of 96%. The blind test confusion matrix is shown in Figure 8.

### 3.2. Recognition of the Ethanol Concentration

**Figure 9:**
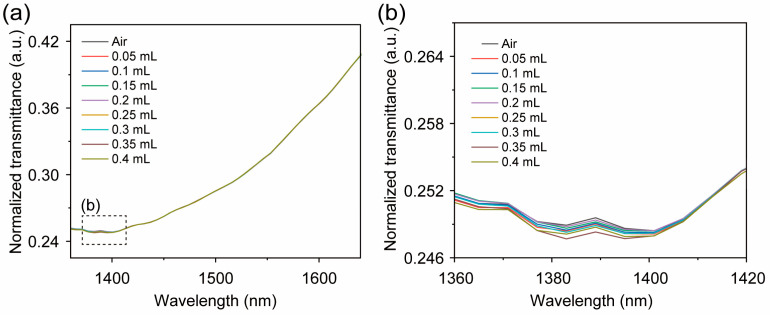
The near infrared spectra of 9 different concentration of the ethanol gas samples. (**a**) The normalized transmittance of 9 different concentration of the ethanol gas samples in the 1350 to 1650 nm wavelength range. (**b**) The normalized transmittance of 9 different concentration of the ethanol gas samples in the 1360 to 1420 nm wavelength range. The air in this test is set as the blank sample; among the 90 ethanol gas samples, we extracted 1 gas sample from each ethanol gas samples to form this near-infrared spectrum.

In the recognition of the ethanol concentration test, we divided the 90 samples of 8 different ethanol concentrations into 63 training set samples and 27 blind test set samples; the normalized spectral information of each gas sample is shown in Figure 9a. Similarly, because the sensitivity of near-infrared spectral gas sensors is relatively low and due to the influence of system noise, although the spectral absorption peak with ethanol gas samples could be observed, and the transmittance is inversely proportional to the ethanol gas samples’ concentrations, it is difficult to distinguish between the same gas samples with small concentration differences, and there are only slight differences in some bands (as shown in Figure 9b). Then, we used normalized spectral preprocessing combined with the quadratic kernel function SVM algorithm (the accuracy of the other machine learning methods is shown in Table 2), which demonstrated a cross-validation accuracy of 92.6% and a blind test accuracy of 96%.

**Figure 10:**
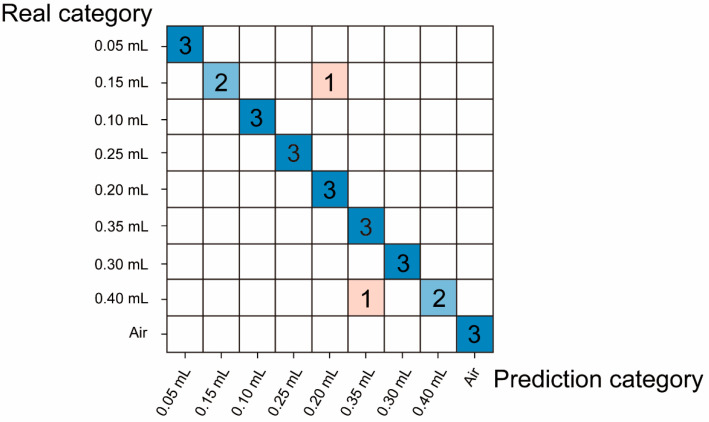
The confusion matrix of the different ethanol gas concentration samples blind test. The blue block indicates that the prediction category is consistent with the real category, and the red block indicates that the prediction category is inconsistent with the real category. The darker the color block, the more correct samples are predicted.

**Figure 11:**
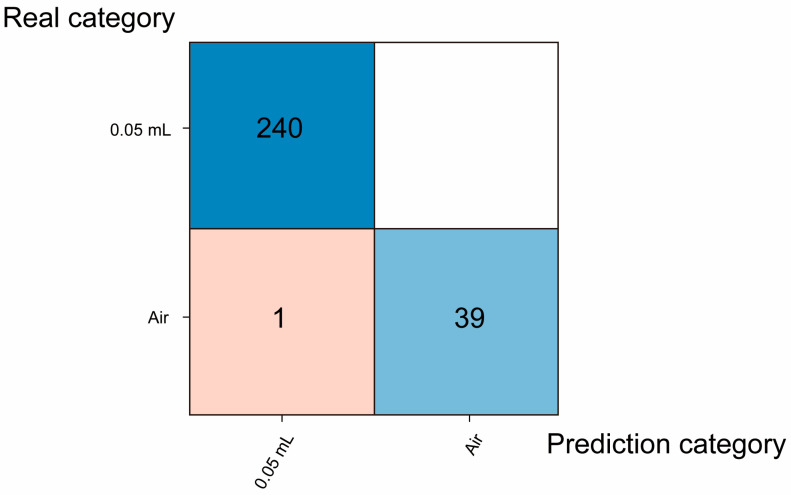
The confusion matrix of the 0.05 mL ethanol gas samples blind test. The blue block indicates that the prediction category is consistent with the real category, and the red block indicates that the prediction category is inconsistent with the real category. The darker the color block, the more correct samples are predicted.

The blind test confusion matrix is shown in Figure 10. Figure 10 shows that air as a blank sample and ethanol of various concentrations could be well distinguished. Then, to test the minimum detection limit of the NIR spectral gas sensor combined with the quadratic kernel function SVM algorithm, we conducted an experiment using 0.05 mL of ethanol volatilized in a 52 L sealed transparent plastic box as the ethanol sample, with air as the blank control. After modeling, we evaluated whether any misclassification occurred between the ethanol and air categories. The test was repeated 280 times, with only a single instance of misidentification at the 369.43 ppm concentration in the wavelength range from 1350 nm to 1650 nm (Figure 11).

Table 3 lists the different gas sensors used for odor recognition. Among these, the combination of the NIR spectral gas sensor and the quadratic kernel function SVM algorithm demonstrates a significant enhancement in detecting the ethanol concentration. This combination not only improves the detection accuracy but also reduces the reliance on multiple sensors for identifying diverse gas samples. By minimizing the number of sensors required, this approach offers a more efficient and cost-effective solution for multi-gas detection.

## 4. Conclusions

In conclusion, we adopted the main method of silicon wafer surface treatment to process the Fabry–Perot cavity and performed a high reflectivity analysis in the operating band. Using three-dimensional stacked packaging to integrate the hardware part, combined with spectral preprocessing and spectral modeling algorithms in the software part, we realized a MEMS NIR spectral gas sensor with a compact folded optical path and an enhanced gas spectral absorption. To demonstrate its reliability and practicality in mixed gases differentiation and gas detection, the MEMS NIR spectral gas sensor was used to simultaneously monitor multiple parameters, including multiple combinations of gas samples of ethanol, Korean kimchi, and durian pulp, as well as ethanol concentrations. The designed microsystem exhibits selective and sensitive detection at room temperature toward multiple combinations of gas samples comprised of ethanol, Korean kimchi, and durian pulp, with a cross-validation accuracy of 91.4% and a blind test accuracy of 96%, and also demonstrates an ethanol detection limit of around 369 ppm (with a cross-validation accuracy of 92.6% and a blind test accuracy of 96%). Additionally, it shows a fast response (within 6 s) with good recyclability. However, the MEMS NIR spectral gas sensor may produce misidentifications when encountering odors outside the existing model library. To address this, we propose using a clustering-based classification method. Odors not included in the library would be categorized as an “unknown odor”. The spectral data for these unknown odors would then be added to the model library, facilitating its continuous expansion. This iterative process would progressively enhance the library, increasing the variety of odors it can recognize. The MEMS NIR spectral gas sensor combined with normalized spectral preprocessing and the quadratic kernel function SVM algorithm outperforms most of the NIR spectral gas sensors. The demonstrated performance enhancement and multi-component detection in food of the MEMS NIR spectral gas sensor and its usage in simultaneous detection of multiple factors provide novel insights for making gas sensors with miniaturized sizes and high performance, which are promising traits for mass production and commercialization.

## Figures and Tables

**Table 1:** The accuracy of different machine learning methods for distinguishing multi-component gas samples.

Classification Algorithm	Accuracy
Decision tree	56.9%
Linear discriminant analysis	75.0%
K-NN	82.8%
SVM	91.4%

**Table 2:** The accuracy of different machine learning methods for the recognition of the ethanol concentration.

Classification Algorithm	Accuracy
Decision tree	73.0%
Linear discriminant analysis	57.1%
K-NN	87.3%
SVM	92.6%

**Table 3:** Odor detection gas sensors.

Sensor Type	Gas Type	Simultaneous Detection of Multiple Gases	Number of Sensors	Accuracy (Highest)	Detection Limit	Reference
Commercial MOS	Air contaminants	Yes (binary gas mixtures)	4	95.7%	N/A	[34]
Chemically resistive gas sensors	Air contaminants	Yes	8	95.14%	N/A	[35]
Chemiresistive odor sensor	Oil gas	Yes	24	93.9%	N/A	[36]
NIR spectral gas sensor	Ethanol	No	1	N/A	1.5%	[37]
NIR spectral gas sensor	Natural gases	Yes	1	95%	1.42% @ 30 s (propane) 1.67% @ 30 s (butane)	[15]
MEMS NIR spectral gas sensor	Ethanol, Korean kimchi, durian pulp	Yes	1	96%	369 ppm @ 5 s (ethanol)	This work

## Data Availability

Data is contained within the article.

## Abbreviations

The following abbreviations are used in this manuscript:

MEMS	Micro electromechanical system
NIR	Near-infrared
FT-IR	Fourier transform infrared
CVD	Chemical vapor deposition
DBR	Distributed Bragg reflector
PECVD	Plasma-enhanced chemical vapor deposition
LPCVD	Low-pressure chemical vapor deposition
CEAS	Cavity-enhanced absorption spectroscopy
SNV	Standard normal variate
SVM	Support vector machine
